# Role of Psychological Contracts in Enhancing Employee Creativity Through Knowledge Sharing: Do Boundary Conditions of Organization’s Socialization and Work-Related Curiosity Matter?

**DOI:** 10.3389/fpsyg.2022.834041

**Published:** 2022-06-14

**Authors:** Boliang Jiang, Tribhuwan Kumar, Nabeel Rehman, Rizwana Hameed, Mehmet Kiziloglu, Adan Israr

**Affiliations:** ^1^Management Innovation and Evaluation Research Center, School of Management, Tianjin University of Commerce, Tianjin, China; ^2^Department of Management Sciences, Prince Sattam Bin Abdulaziz University, Al-Kharj, Saudi Arabia; ^3^Department of Management Sciences, Lahore Garrison University, Lahore, Pakistan; ^4^Department of Management Sciences, The University of Lahore, Lahore, Pakistan; ^5^Institute of Business Management, University of Engineering and Technology, Lahore, Pakistan; ^6^Management and Organization Department, Pamukkale University, Denizli, Turkey; ^7^Department of Psychology, Government College University, Faisalabad, Pakistan

**Keywords:** psychological contracts, employee creativity performance, knowledge sharing, human resource management (general), work-related curiosity

## Abstract

COVID-19 has had a huge impact on workers and workplaces across the world while putting regular work practices into disarray. Apart from the obvious effects of COVID-19, the pandemic is anticipated to have a variety of social–psychological, health-related, and economic implications for individuals at work. Despite extensive research on psychological contracts and knowledge sharing, these domains of pedagogic endeavor have received relatively little attention in the context of employee creativity subjected to the boundary conditions of the organization’s socialization and work-related curiosity. This study investigates, empirically, the role of psychological contracts in escalating employee creativity through knowledge sharing by considering the moderating role of an organization’s socialization and work-related curiosity. The response received from 372 employees of the manufacturing sector has been investigated and analyzed through Smart PLS software. The results have revealed that knowledge sharing is mediating the relationship between psychological contract and employee creative performance, whereas the moderators significantly moderate the relationships between psychological contract and knowledge sharing and between knowledge sharing and employee creative performance accordingly. It has also been depicted that the moderating impact shown by both moderators is significantly high.

## Introduction

Following COVID-19, the entire world was confronted with a tragic and unprecedented scenario that disrupted all aspects of human life. Lockdown and social isolation were among the measures employed to slow down the spread of the pandemic, which eventually led to the shutdown of companies (Karim et al., 2020). The novel COVID-19 epidemic in 2020 has also completely affected people’s work patterns. Organizations have experienced difficulty in enabling workers to perform successfully and in sustaining their job engagement throughout the interruption due to the unprecedented global crisis (Gallup, 2020). Individual employees’ active engagement in combating such a crisis is as vital as the companies’ attempt to deal with it by adapting to the new environment and cultivating creativity.

To survive in today’s dynamic business environment and remain competitive, organizations need to remain innovative and creative in their business processes (Li and Merchant, 2021). Creativeness can only be achieved if an organization acts, in an informed manner, to enhance its employees’ creative performance. In this concern, one of the fundamental elements, which fosters employee creativity, is the psychological contract; it determines the level of efforts exerted by the employees in the form of knowledge sharing (Landry et al., 2014; Luu, 2016), and these efforts can be observed in extra-role behavior of the employees. In such a case, the performance of employees normally exceeds the formal roles and responsibilities that are assigned to them by their employers. On the contrary, in the absence of a psychological contract, as reflected in the social exchange theory (Robinson and Rousseau, 1994), the employees have less inclination to surpass their self-interests and make honest efforts for sharing their skills, knowledge, and expertise with their peers (Gudergan and Lings, 2010).

Therefore, an organization needs to formulate a strong basis for psychological contract fulfillment. In this way, the employees will probably develop an attitude of knowledge sharing and will lead their firm to endeavor successful ventures and stay competitive (Rousseau, 2004; Kremer et al., 2019). The employees with the psychological contract are voluntarily involved in skill-developing activities, have a high potential to gain new knowledge, develop knowledge-sharing attitude, and intend to improve their critical thinking skills (Zhang and Bartol, 2010); this ultimately improves their creative performance (Liao and Chen, 2018). Knowledge sharing is a fundamental tool that encompasses various knowledge management activities through which employees can effectively apply the knowledge and enhance their creative performance, which enables the firms to achieve a sustainable competitive edge (Runco, 2008).

The knowledge-sharing behaviors are of immense importance as highlighted by recent studies (Usman et al., 2019; Anser et al., 2020); however, because of its complex nature (Park et al., 2004), the firms find it challenging to devise different ways to promote sharing knowledge among employees (Kremer et al., 2019). In this regard, it is essential to analyze antecedents of knowledge sharing, such as psychological contract fulfillment, which make the employees believe that they are valued enough to be receiving all of the promised benefits from their employers, thereby enabling them to take the initiatives for knowledge sharing (Dixon-Fowler et al., 2020).

The SMEs (small and medium enterprises) sector of any economy has an important and strategic role in national economic development. Scholars have suggested that there is a need to emphasize the importance of knowledge sharing and creative performance in the SMEs sector (Setyanti and Farida, 2016). In big and multinational corporations, non-tangible assets, such as ideas, new information, and knowledge, are emphasized as these are considered an important source for innovation and firm performance (Teece, 2014). Hence, these organizations must actively promote knowledge sharing among employees to yield valuable outcomes (Hameed et al., 2019). However, SMEs, which are the backbone of the economy of developing countries, struggle to devise HR policies and utilize the concept of knowledge sharing as a strategic tool to achieve innovation and creativity. Furthermore, SMEs received less attention from the researchers in this particular context. This study addresses the situation and considered a sample of 372 employees from the manufacturing sector of SMEs in Pakistan, with an aim to provide vital practical knowledge that may prove helpful for the management in decision-making and devising HR strategies to enhance employee creativity through the physiological contract and knowledge sharing.

The researchers argue that the knowledge-sharing behavior is embedded in social ties between the employees and socialization activities initiated by firms (Kim and Moon, 2021), mainly by human resources to promote knowledge sharing (Zhang and Jiang, 2015). Organizational socialization is “the process by which an individual acquires the social knowledge and skills that are necessary to assume an organizational role” (Van Maanen et al., 1979). Hence, it is argued that the creativity of employees is highly affected by knowledge sharing, and here, psychological contract plays an important role in determining this creativity performance with the utilization of boundary conditions of socialization tactics.

In addition, work-related curiosity is considered as a self-assessment tool for an individual to perform well in job-related activities, as it gives a desire to gain new knowledge, convert knowledge into workable ideas with novelty, and let an employee exercise extra-role activity. Curious people possess a desire for learning (Mussel, 2010) and exert their efforts in the self-regulation process to engage themselves in complex reasoning and problem-solving (Schmeichel et al., 2003; Schutte and Malouff, 2020), which ultimately promote creativity (Schutte and Malouff, 2020). The literature suggests that work-related curiosity is initiated because of the high level of psychological contract fulfillment (Niesen et al., 2018).

Previously, the employee’s creative behavior, as an outcome, has been ignored in the field of psychological contract research. However, an understanding of the mechanism through which the psychological contract affects employee creativity performance with the intervening role of knowledge sharing is not clear (Nnaji-Ihedinmah et al., 2020). Furthermore, Hameed et al. (2019), while highlighting the importance of knowledge sharing in their study, posed a dire need to develop the underlying mechanisms to explain how knowledge sharing can generate competitiveness for an organization. To address this research gap, this study contributes to the literature by (1) empirically investigating the indirect impact of psychological contract on employee creative performance through knowledge sharing, (2) studying the moderating role of an organization’s socialization tactics in the relationship between psychological contract and knowledge sharing, and (3) studying the moderating role of work-related curiosity in the relationship between knowledge sharing and employee’s creative performance. The objective of the study was to develop a theoretical framework to investigate mediation and moderation among variables as discussed earlier. Furthermore, the findings of this study suggested a positive impact of psychological contracts on employee creativity through knowledge sharing. Furthermore, the moderating effect of an organization’s socialization tactics and work-related curiosity has also been found significant. Detailed theoretical as well as practical implications have been discussed in the final section of this study. The findings will substantially educate and empower the management, policymakers, and future researchers.

## Literature Review and Theoretical Framework

**FIGURE 1:**
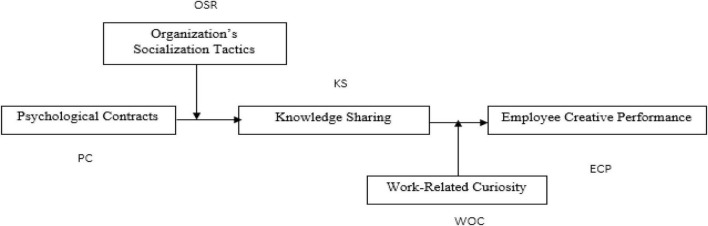
Theoretical model.

The current framework of the study comprises five variables. The interrelationship of these variables has been investigated in a way that psychological contracts have been treated as an independent variable and employee creative performance as the dependent variable with the mediating role of knowledge sharing between the relationship of these variables. In addition to that, the moderating role of an organization’s socialization tactics in the relationship between psychological contracts and knowledge sharing and the moderating role of work-related curiosity in the relationship between knowledge sharing and employee creative performance have been investigated empirically. The theoretical model is presented in Figure 1.

### Psychological Contract and Knowledge Sharing

This study sees psychological contract under the light of social exchange theory (SET). The SET (Blau, 1964) explains that the relationship between a superior and a subordinate is reciprocal. It is believed that, if an employee receives admiration, recognition, and support from the organization, he/she will extend the support back to the organization, which can be observed in his/her extra-role behaviors at the workplace. Studies have suggested that high-quality social exchange relationships can result in multiple employee outcomes by shaping the employee beliefs, i.e., organization citizenship behavior, commitment, and loyalty (Madsen et al., 2005; Bernerth et al., 2007). Furthermore, a recent study has suggested that such social exchange relationships explain underlying mechanisms through which an organization can shape employee negative responses, e.g., an organization’s supportive behavior helps in building high-quality superior–subordinate exchange relationships which, ultimately, strengthen employees’ belief in the organization and discourage any negative reactions during times of change implementation (Rehman et al., 2021a). Accordingly, it can be argued that high-quality social exchange relationships encourage employee psychological contracts. Similarly, Rousseau (1998) has explained psychological contracts as the beliefs of an employee, about himself/herself and his/her employer, regarding the terms and conditions of its reciprocal exchange agreement.

After a deep review of the literature, it can be argued that organizational creativity is the key to an organization’s ongoing sustainability and competitive advantage. As the business environments are more dynamic than ever before, the organizations need to constantly innovate and stay creative to remain on the competitive edge. Hence, creativity is relatively important and an organization’s creativity is determined by its employees’ creative performance. This study identifies that employee psychological contract is a fundamental element that fosters employee creativity indirectly through knowledge sharing. Further, the buffering effect of an organization’s socialization and work-related curiosity has also been empirically investigated.

Adopting new ways to enhance current knowledge and being involved in the creation of new knowledge can have a great impact on the playing field of the employees in the context of an organization where they work (Bankins et al., 2020). The changes in the psychological contract can make employees believe that certain constituents, related to their psychological contracts, have been compromised.

Concerning knowledge sharing, if the pre-employment perspective is considered, then the interview questions must demonstrate experience representing transferable abilities that prefer to help ease knowledge sharing (Mohammed and Dumville, 2001). On the part of organizations, accurate information must be provided so that applicants may be able to judge their fit with the organization’s culture (Schein, 1999). The applicant relies on different sources such as corporate websites, articles published on the Internet, or those published in business or trade magazines, for example, fortune magazines and top businesses survey job expos and on-campus job fairs to gather the corporate information. Afterward, upon hiring, employees should further be supported to develop expectations regarding knowledge sharing because the consistency between a firm’s values and knowledge-sharing behavior can empower candidates to precisely picture organizations’ prospects (Cable et al., 2000). A combined effort from an employee and an employer is valuable in strengthening a firm’s image as a knowledge-based entity (Abdinnour-Helm et al., 2003).

**H1**: Psychological contract has a significant positive impact on knowledge-sharing behavior.

### Knowledge Sharing and Employee Creativity

Individuals’ active information or sharing of knowledge is far more important in a crisis event since it enables firms to resolve issues more efficiently and quickly (Kim and Rhee, 2011); moreover, it encourages businesses to be innovative (Tulshyan, 2020). As a result, solutions to ensure open knowledge exchange among personnel during a crisis must be developed (Lee et al., 2020). Knowledge sharing among employees serves as a tool (Wynder, 2007; Kessel et al., 2012) for individuals to promote diverse knowledge within their workplace as it enhances creativity within an organization (Perry-Smith, 2006). Therefore, individuals consider knowledge as an important asset. Sung and Choi (2012) argued that a teamwork environment results in the diversification of approaches and that it provides more opportunities to mix the existing information and ideas in a better way. The researchers are of the agreement that creativity can be higher if the team encourages distributing relevant knowledge among its members (Gino et al., 2010; Griffith and Sawyer, 2010). Knowledge sharing leads to a more comprehensive thought pattern, which is important for empowering collective creativity. Different departmental teams, such as R and D, manufacturing units, and operational management, have been observed to be supported by the impact of association of knowledge sharing and creativity.

**H2**: Knowledge sharing has a significant positive impact on employee creative behavior.

### Mediation Effect of Knowledge Sharing Between Psychological Contract and Employee Creative Performance

In view of its certain nature, an effective psychological contract includes acknowledgment of its reciprocity and commonality of contract (Koh et al., 2004). Bock et al. (2005) designed a measure for empirically investigating knowledge sharing with other variables, which is helpful for scholars. For instance, one item from the scale was asking the individual how worthy their experience with others was regarding knowledge sharing and afterward giving a behavioral example to show their opinion regarding ratings. Tsai et al. (2015) proposed that, for a business environment to enhance encouragement among employees and increase their creativity, four dimensions such as knowledge sharing, motivation, procedural justice, and promotions are considered the most important factors for any organization. To avoid the risk of failure and uncertainty, employees should be more focused and should apply extra effort and share maximum knowledge to create a knowledge network that can positively affect creativity (Handy et al., 2020).

**H3**: Psychological contract has an indirect significant positive impact on employee creativity mediated by knowledge sharing.

### Moderating Effect of Organization’s Socialization on Psychological Contract and Knowledge Sharing

To provide support to psychological contracts, structured socialization processes can be as effective as informal socialization techniques (Robinson and Wolfe Morrison, 2000; Cooper-Thomas et al., 2012). Among traditional techniques for the fulfillment of an organization’s responsibilities such as the orientation of new entrants and indulging dynamism among current workers, expert databases, knowledge-based intranets, joint workplaces, and communication techniques can be found to be very effective and may serve the purpose of encouraging knowledge sharing (Wu and Yan, 2020). The interpersonal needs of employees are met through socialization tactics that enhance trust through more interaction among employees and have a positive effect on the level of psychological contract perceptions. In this regard, mentoring projects can be utilized to coordinate with employees to have a positive impression of the psychological contract. These formal mentoring programs are not as compelling as those of informal and casual ones made, which can be planned for employees to have more work-related interaction (Ragins and Cotton, 1999; Ragins et al., 2000). Providing more chances of socializing to existing and new workers can prove to be very effective and may increase commitment, which further builds up confidence among employees to share knowledge.

Reinforcement of psychological contract perceptions and their realignment through interactive sessions with employees allows them to focus on various activities that enhance the available networking opportunities within the workplace (Newaz et al., 2020). It also establishes more familiarization with workplace systems, and as a result, knowledge sharing grows among current as well as new employees. Such knowledge-sharing activities enhance trust between employees and increase the chances that a newcomer joining such a culture will feel himself/herself a worthy person because the knowledge is being shared with him/her and in return, he/she becomes passionate to gain more knowledge and feels the desire to share it in return (Tsai et al., 2015).

**H4**: Organization’s socialization tactics positively moderate the relationship between psychological contract and knowledge sharing.

### Moderating Effect of Work-Place Curiosity on the Relationship Between Knowledge Sharing and Employee Creativity

Social exchange theory proposes that an individual’s actions are conditional on a return or reward (Blau, 1964). The norm of reciprocity in social exchange theory suggests that individuals are keener to share knowledge according to their reciprocal cost, benefit analysis, and their self-interest (Cabrera and Cabrera, 2005). Knowledge sharing in a workplace environment can be related to any form such as individual work experience, working procedures, and any kind of official documentation and procedures (Lu et al., 2006). Woodman et al. (1993) explained that creativity is something that has been intuited in one’s mind and is about gathering ideas. Choi (2004) described that creative ability is directly related to an individual’s perspective to see a problem from a different dimension and utilize the necessary skills to solve it; therefore, this attitude allows an individual to exhibit creative performance.

Employees with a high score on curiosity have better adaptability (Harrison et al., 2011) as they are keen on gaining knowledge and learning through socializing in the work environment (Reio and Wiswell, 2000). The work-related curiosity is a positive indicator of one’s self-assessment toward his job performance and career success. Curiosity has widely been defined as the desire of gaining new knowledge and experiences to elaborate exploratory behavior (Loewenstein, 1994). As an individual difference, curiosity is depicted as a thirst for acquiring knowledge by either developing new ideas, solving problems, or gaining knowledge about intellectual ideas regarding different situations (Gruber et al., 2019).

**H5**: Work-related curiosity positively moderates the relationship between knowledge sharing and employee creative behavior.

## Methodology

### Measures

The construct’s items were measured by using a Likert scale with seven points ranging from 1 = strongly disagree to 7 = strongly agree. The psychological contract was measured by using a four-item scale of global measure adapted from Conway and Briner (2002). The knowledge-sharing items were adapted by using the five-item scale of Chow and Chan (2008). The employee’s perspectives according to the creativity questionnaire developed and used in prior research by George and Zhou (2001) were adapted with a 13-item scale as self-reports to measure employee creativity. The socialization tactics were investigated by taking six items based on the study of Cable and Parsons (2001). Finally, work-related curiosity was operationalized by using 10 items from Mussel (2013).

### Sample and Procedure

Manufacturing sector medium-sized SMEs were observed as the population of this study. Based on the definition given by the small and medium enterprises development authority (SMEDA), those firms that have more than 50 employees but equal to or fewer than 250 employees are considered medium-sized SMEs. A list of employees from medium-sized firms was developed out of registered medium-sized firms in SMEDA. In our study, employees in SMEs working under higher-level managers were the respondents.

This study applied stratified random sampling based on seven major cities of Punjab province (Gujranwala, Lahore, Sialkot, Gujrat, Multan, Sheikhupura, and Faisalabad) that were selected for the study as used by Nabeel-Rehman and Nazri (2019). These cities were selected based on the maximum number of industries and hosting 65% of all of the industries in Punjab (Rehman et al., 2020; Rehman et al., 2021b). These firms include leather, sports, textile, metal and wood, furniture, food and beverages, and others, and 57% of the industry was above the age of 5 years. A total of 1,200 questionnaires were distributed through courier/self-presented and services of survey firms to attain a high response rate. The declaration was given to respondents about their responses confidentially. We received a total of 398 responses with a percentage of 33.16%, but 26 responses were not complete; therefore, for the final dataset, a total of 372 responses were used. Table 1 presents the sample distribution summary by industry.

**TABLE 1:** Sample distribution by industry.

Industry	No. of firms	Percentage	Questionnaire distributed	Questionnaire received
Textile	1,811	21	278	91
Leather	1,207	14	152	59
Sports	1,034	12	116	48
Food and beverages	1,638	19	242	68
Metal	690	08	94	31
Wood and furniture	823	10	130	65
Others	1,380	16	188	46
Total	8,623	100	1,200	398

To reduce the non-response bias, this study compared the characteristics of non-responding firms with responding firms. Furthermore, to check whether the sample of this study is representing the target population, this study has matched the sample mean for descriptive characteristics of selected firms and the target population. The findings t-statistics highlight that there was no statistically significant difference between non-responders and respondents or between the target population and the sample population. Furthermore, because all of the measurements in this study were obtained using the same survey instruments, we used Harman’s test to avoid common method bias.

### The Measurement Model

The correlation values among those factors should not be higher than 0.85 (Kline, 2005). The values on the arrows that are linking factors to items represent the standardized factor loadings. The independent variable, psychological contract loadings, are within the range and are represented by four items, while the dependent variable as employee creative performance has 13 items with an acceptable item loading range. The mediator, represented by five items, loading range is acceptable. The socialization tactics have six items, and work-related curiosity has seven items. The scores of factor loadings were greater than the 0.70 baselines.

Smart PLS 3.2.6 is used to examine the research framework because it facilitates the non-normality of the sample size. This research evaluated the construct reliability with PLS’s internal consistency measure. This study also analyzed convergent validity by investigating the AVE from the measures. AVE highlights the complete variance of indicators explained by a latent construct (Fornell and Larcker, 1981). The values of AVE in this study are above the threshold value which is 0.50. Indicator and composite reliability (CR) are measured, whereas threshold values for the indicator reliability are 0.70 (Gliem and Gliem, 2003). The values of construct reliability, CR, AVE, and factor loading are presented in Table 2.

**TABLE 2:** Reliability and validity.

	Cronbach’s alpha	CR	AVE	Factor loadings
ECP	0.939	0.947	0.580	0.711–0.838
KS	0.864	0.902	0.650	0.745–0.891
WOC	0.923	0.939	0.687	0.754–0.911
OSR	0.890	0.916	0.648	0.733–0.884
PC	0.881	0.918	0.737	0.816–0.908

This study evaluated the values of the Heterotrait–Monotrait (HTMT) ratio to analyze the discriminant validity. The HTMT ratios results presented in Table 3 show that the values are below the threshold of 0.85 and recommend the reflective constructs’ discriminant validity (Petter et al., 2007).

**TABLE 3:** Heterotrait–Monotrait (HTMT) ratio.

	ECP	KS	OSR	PC	WOC
ECP					
KS	0.513				
OSR	0.319	0.448			
PC	0.351	0.589	0.415		
WOC	0.564	0.530	0.402	0.489	

### Structural Model

**FIGURE 2:**
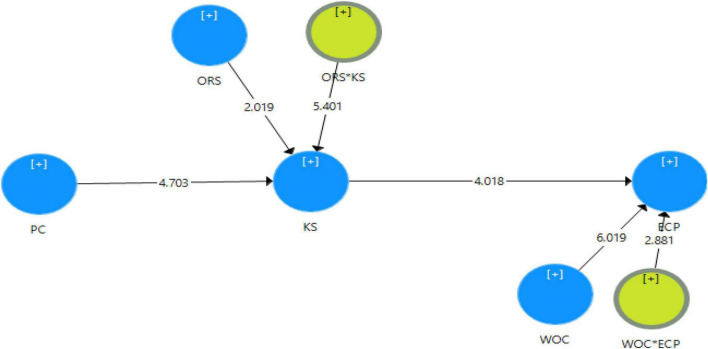
PLS-SEM model with indirect and direct effects.

After the appropriate and acceptable results of the measurement model, this study analyzes the research hypotheses by utilizing the PLS-SEM. There were five research hypotheses, and all hypotheses of this research are supported. The PLS structural model is presented in Figure 2.

The results show that the PC has a significant influence on KS (β = 0.280, *p* < 0.000). Similarly, KS has a significant influence on ECP (β = 0.198, *p* < 0.000). This study also analyzes the indirect effect of PC on the ECP of SMEs through KS. The results of the specific indirect path demonstrate that KS mediates the relationship between PC and ECP (β = 0.055, *p* < 0.003).

The results of the moderation analysis present that organization’s socialization tactics (OSR) significantly moderate the relationship between PC and KS (β = 0.157, *p* < 0.003). The results of another moderator present that work-related curiosity also has a moderating effect on KS and ECP (β = 0.095, *p* < 0.003). Similarly, WOC (work curiosity) significantly moderates the relationship between PC and KS (β = 0.095, *p* < 0.003). OSR*KS and WOC*ECP show the moderation effect and the results of the path coefficient are presented in Table 4.

**TABLE 4:** Path coefficients with *P*-values.

	Path coefficient	T-statistics	*P*-values
KS → ECP	0.198	4.117	0.000
OSR → KS	0.102	2.085	0.038
OSR*KS → KS	0.157	5.470	0.000
PC → KS	0.280	4.790	0.000
WOC → ECP	0.322	5.629	0.000
WOC × ECP → ECP	0.095	2.982	0.003
PC → KS → ECP	0.055	2.975	0.003

### The Goodness of Fit

Although the goodness of fit (GoF) indices cannot be generated by the PLS-SEM, the model’s explanatory power can be evaluated by considering the *R*^2^-value (Henseler et al., 2015). To assess the fitness of the model, a diagnostic tool developed by Tenenhaus et al. (2005) is used as the GoF index for PLS-SEM. This GoF is calculated by taking the geometric mean of AVE values and the average values of *R*^2^. By following the guidelines of Henseler et al. (2015), this study measures the GoF as shown in Table 5.

**TABLE 5:** Goodness of fit.

Constructs	AVE	*R* ^2^
PC	0.737	
OSR	0.648	
KS	0.650	0.383
WOC	0.687	
ECP	0.580	0.366
**Average score**	0.660	0.374

AVE × R^2^	0.660 × 0.374 = 0.247	
**GoF** = √(*AVE*×*R*^2^)	**0.496**	

The GoF score in this study is 0.496, indicating that the PLS model is fit. It demonstrates that the theoretical model is capable of accounting for 49.6% of the possible fit, indicating that the model is adequate. This depicts that the variables in the theoretical model are better able to explain the interrelationships concerning their direct, indirect, and moderating impact.

All direct relation path hypotheses (H1 and H2) are significant and have a positive relationship. The results of the meditation analyses show that knowledge sharing has an intervening role between psychological contract and employee creativity. The moderating effect of the two moderators in H4 and H5 shows that they are significantly moderating the relationships, which depicts that socialization tactics and work-related curiosity should be considered while expecting a creative performance from employees. Table 6 present the results of the hypotheses.

**TABLE 6:** Hypotheses results.

Hypothesized path (hypothesis)		Beta value	*T*-value	*P*-value	Supported
H1: PC → KS	The psychological contract has a significant positive impact on knowledge-sharing behavior	0.280	4.790	0.000	YES
H2: KS → ECP	Knowledge sharing has a significant positive impact on employee creative behavior	0.198	4.117	0.000	YES
H3: PC → KS → ECP	The psychological contract has an indirect significant positive impact on employee creativity mediated by knowledge sharing	0.055	2.975	0.003	YES
H4: OSR × KS → KS	An organization’s socialization tactics positively moderate the relationship between psychological contract and knowledge sharing	0.157	5.470	0.000	YES
H5: WOC × ECP → ECP	Work-related curiosity positively moderates the relationship between knowledge sharing and employee creative behavior	0.095	2.982	0.003	YES

## Discussion and Conclusion

Considering the prominence of psychological contracts in the domain of knowledge sharing, this study contributes to the literature by enhancing our understanding of the role that these relationships play in achieving employee creative performance. This study achieves this purpose by observing the impact of moderating mechanisms, specifically work-related curiosity, on the interplay of relationships where the psychological contract, as an antecedent, affects employee creativity indirectly through knowledge sharing. This research augments our understanding in the domain of psychological contracts and knowledge sharing to understand the employee’s creative behavior, whereas the strength of the two relationships, path-wise from psychological contracts to knowledge sharing and knowledge sharing to employee creative behavior is moderated by the organization’s socialization and work-related curiosity. Encompassing previous studies, the current research work enhances the understanding of these relationships by providing a clear picture of knowledge sharing as an imperative tool that can prevail in the presence of psychological contract fulfillment, which motivates employees to be more engaged in creative behavior.

The empirical evidence of this study suggests that an employee’s psychological contract positively impacts knowledge-sharing behavior. These findings converge with the previous literature (Abdinnour-Helm et al., 2003; Bankins et al., 2020). The relationship between knowledge sharing and employee creativity is significantly positive. Furthermore, the psychological contract indirectly enhances employee creativity through knowledge sharing. This supports the previous literature (Wynder, 2007; Kessel et al., 2012). Although some of the previous studies considered knowledge sharing, work-related curiosity, and innovation, most of the time, the main focus of the researchers remained on multinational corporations (Hameed et al., 2019; Shahzad et al., 2020); hence, contextual differences between a multinational organization and SMEs (particularly of a developing country) remained ignored. This study took a comprehensive approach and contributed to the theory by empirically investigating the relationships between psychological contract, knowledge sharing, and employee’s creative performance in the context of SMEs in a developing country. Furthermore, the mediating role of knowledge sharing has also been emphasized under the buffering effect of the organization’s socialization tactics and work-related curiosity. The findings suggest the positive impact of both on subsequent relationships of psychological contract and knowledge sharing as well as knowledge sharing and employee creativity. The model developed in this study will help in devising HR policies and strategic adaptations of psychological contracts and knowledge sharing to ultimately boost employees’ creative performance.

### Practical Implications

The managers in SMEs should take employees into confidence regarding any breach of psychological contract fulfillment, especially when employees are fulfilling their obligations toward their employers; this active role of managers would help in promoting positivity at the workplace and motivating employees to engage in their work beyond formal requirements. As a result, employees tend to be more creative through sharing knowledge and building on their existing knowledge domains. This will result in increased trust in management and enable employees to enhance their creative performance through creative idea generation and be innovative to add more value to their work. Although employees hold expectations of the fulfillment of the psychological contract, at the same time, the culture of open communication can greatly help in setting up the relationship between employees and the organization to better understand the expectations from both sides. Dries and De Gieter (2014) are of the view that organizations should monitor the behavior patterns of employees to fulfill expectations of their high potential employees on an ongoing basis so that these individuals can be retained in organizations, do their best, and succeed in their career with more intellectual capabilities.

The management should be specially trained to make realistic promises to the employees which can be fulfilled in future. In this regard, the HR practices should primarily be focused on pre-job and post-job activities such as providing realistic job descriptions during interviews and the selection processes and incorporating such questions during the whole job period that assess employee expectations with sophistication and also explain to them what is expected from them by their employer. Newly hired and current employees should be monitored by their supervisors and HR managerial staff by keeping the perceptions and promises into consideration. Also, they should be given opportunities to socialize with peers and those working with more experience to share their expertise, which will surely escalate the commitment and contribution of employees toward their organization.

Management should be vigilant and aware of the perspective of observing any incongruence in case of unmet expectations, especially when supervisors or managers are changed or replaced with new managers. The new managers should be informed about the existing employee’s expertise and contributions as well as about the expectation to keep their role aligned with employees’ formal expectations to allow employees to perform with their best skills and expertise even with the new supervision. In this regard, Eilam-Shamir and Yaakobi (2014) are of the view that employee expectations are always on the rise; therefore, it is crucial to specify the design of job orientation sessions and familiarization of workplace practices when the employees are assigned with the job responsibilities so that the gap in expectation continuum should be minimized. In this way, employers can improve employees’ outcomes by better managing their psychological contract and making realistic promises that further lead to improving employees’ retention and commitment with positive outcomes.

Last but not the least, workers’ inherent needs are more likely to be satisfied when firms provide considerable information about the crisis (e.g., COVID-19), invite employees to determine the information they need, and provide both positive and negative aspects of the crisis of the company. Knowledge exchange, particularly through personal interaction, has long been seen to increase the likelihood of corporate innovation and creativity.

### Limitations and Future Recommendations

This study is prone to a few limitations, but it also offers new horizons for future researchers. The data collection is cross-sectional; hence, considering a longitudinal approach will contribute further toward the literature. Given that longitudinal studies are more sophisticated and can provide in-depth data to better evaluate the employee–employer relations in different contexts unfolding micro-level changes in the psychological contracts, for example, “willingness to contribute.” There are certain factors such as personality, future retirement options, and a work–family balance which may result in a shift in the psychological contract of employees. Future researchers are encouraged to explore these factors. The similar nature of studies may be replicated by analyzing the results in cross-country comparisons. In addition, our study focused on psychological contacts in general rather than explaining them by dimensions. Furthermore, considering the dynamic nature of psychological contacts, a qualitative research approach must be conducted with a retrospective approach. Furthermore, the importance of contexts is unquestionable because of the increasing trend of hiring a diverse workforce. The context perspective can also be studied by conducting field and experimental studies that also underscores the importance of changing the nature of contracts among different age groups. Also, social cognitive theories must be explained in connection with psychological contracts to understand the complexity of behaviors that are intertwined in the employee–employer relationship. The study can be extended by considering employee, group-level, or organizational-level psychological contracts emphasizing the transactional and relational aspects. The aforementioned future directions have many ideas for research scholars in this field to carry out studies with novel ideas by applying various theories and extending methodologies which will help scholars in contributing practical implications. Future studies by scholars will serve as guidance for effective management of these psychological contracts, which will also broaden psychological contracts’ horizon in understanding its implications in different occupations such as engineering, IT, business, and product development.

## Data Availability Statement

The data analyzed in this study is subject to the following licenses/restrictions: As it is confidential data that is provided by the respondents, and as per the restriction ethical committee, data sets is not included. Requests to access these datasets should be directed to ORIC@lgu.edu.pk.

## Ethics Statement

Ethical review and approval was not required for the study on human participants in accordance with the local legislation and institutional requirements. Written informed consent from the patients/participants was not required to participate in this study in accordance with the national legislation and the institutional requirements.

## Author Contributions

BJ: improve the writeup and analysis discussion. TK: take a critical review and incorporate the comments. NR: develop the theoretical model and run the analysis. RH: literature writing and manuscript proofreading. MK: analyzes the data and writes the conclusion part of the study. AI: collect the data. All authors contributed to the article and approved the submitted version.

## Conflict of Interest

The authors declare that the research was conducted in the absence of any commercial or financial relationships that could be construed as a potential conflict of interest.

## Publisher’s Note

All claims expressed in this article are solely those of the authors and do not necessarily represent those of their affiliated organizations, or those of the publisher, the editors and the reviewers. Any product that may be evaluated in this article, or claim that may be made by its manufacturer, is not guaranteed or endorsed by the publisher.
